# Stretchable, Weavable Coiled Carbon Nanotube/MnO_2_/Polymer Fiber Solid-State Supercapacitors

**DOI:** 10.1038/srep09387

**Published:** 2015-03-23

**Authors:** Changsoon Choi, Shi Hyeong Kim, Hyeon Jun Sim, Jae Ah Lee, A Young Choi, Youn Tae Kim, Xavier Lepró, Geoffrey M. Spinks, Ray H. Baughman, Seon Jeong Kim

**Affiliations:** 1 Center for Bio-Artificial Muscle and Department of Biomedical Engineering, Hanyang University, Seoul 133-791, Korea; 2 IT Fusion Technology Research Center and Department of IT Fusion Technology, Chosun University, Gwangju 501-759, Korea; 3 The Alan G. MacDiarmid NanoTech Institute, University of Texas at Dallas, Richardson, TX 75083, USA; 4 Intelligent Polymer Research Institute, ARC Centre of Excellence for Electromaterials Science, University of Wollongong, Wollongong, NSW 2522, Australia

## Abstract

Fiber and yarn supercapacitors that are elastomerically deformable without performance loss are sought for such applications as power sources for wearable electronics, micro-devices, and implantable medical devices. Previously reported yarn and fiber supercapacitors are expensive to fabricate, difficult to upscale, or non-stretchable, which limits possible use. The elastomeric electrodes of the present solid-state supercapacitors are made by using giant inserted twist to coil a nylon sewing thread that is helically wrapped with a carbon nanotube sheet, and then electrochemically depositing pseudocapacitive MnO_2_ nanofibers. These solid-state supercapacitors decrease capacitance by less than 15% when reversibly stretched by 150% in the fiber direction, and largely retain capacitance while being cyclically stretched during charge and discharge. The maximum linear and areal capacitances (based on active materials) and areal energy storage and power densities (based on overall supercapacitor dimensions) are high (5.4 mF/cm, 40.9 mF/cm^2^, 2.6 μWh/cm^2^ and 66.9 μW/cm^2^, respectively), despite the engineered superelasticity of the fiber supercapacitor. Retention of supercapacitor performance during large strain (50%) elastic deformation is demonstrated for supercapacitors incorporated into the wristband of a glove.

Yarn-based micro supercapacitors are attractive power sources for wearable electronics, micro-devices, and implantable medical devices^1^. Various conductive fibers or fibers have been proposed as electrodes for supercapacitors^2^,^3^,^4^,^5^,^6^,^7^. However, scalability, high cost, and low stretchability are problems that can restrict their applications. For example, commercial available metal wires having high electrical conductivity has been used as electrodes for fiber supercapacitor^2^,^3^, but their inborn rigidity restrict their use in textiles to store energy for wearable electronics. Twist-spun carbon nanotube (CNT) fibers can provide high performance as flexible supercapacitor electrodes, due to its high porosity, high surface area, and outstanding mechanical properties^4^,^5^, but CNT fibers are expensive^8^ and break at below 8% strain^9^. Wet-spun graphene/CNT composite fibers provide highly enhanced specific capacitances due to component synergy^6^,^7^, but contain costly carbon single walled nanotubes^7^ and break at ~10% strain^6^. Such limited stretchability of these variously proposed fiber electrodes for supercapacitors may restrict use in advanced applications, like effective power sources for artificial muscles^10^ or highly stretchable electronics^11^. Although buckled^12^ and spring fiber^13^ electrodes have been effectively deployed for highly stretchable film/fiber type energy storage devices, realizing high stretchability, low cost, and scalability for one-dimensional fiber and fiber supercapacitors remain still challenging.

## Results

### Preparation of MnO_2_/CNT/nylon fiber supercapacitor

The fabrication of our fiber supercapacitors starts with strong, low-cost (~$5/kg)^8^, 102 μm diameter nylon fiber (Fig. S1a) used for sewing thread, which is commercially available in effectively unlimited lengths and requires no pre-treatment prior to use. Carbon nanotube aerogel sheet ribbons, which were continuously drawn from a carbon multiwalled nanotube (MWNT) forest^9^, were helically wrapped around the nylon fibers to provide the electronically active fiber electrode component (Supplementary Fig. S1b). Ethanol was subsequently dropped on the fiber surface to produce surface-tension-based densification of the nanotube sheets on the nylon fiber during ethanol evaporation. The resulting binding of the CNTs to the nylon fiber is so strong that the CNT/nylon fiber can be tightly knotted (Supplementary Fig. S1c) without producing any noticeable delamination of the CNTs. This strong bonding results from the densification process and from the small diameter of the nylon fiber (102 μm) with respect to the CNT bundle length, which is much longer than the CNT length (approximately equal to the ~300 μm CNT forest height). Since the CNT bundle length is so much larger that the nylon fiber length, each CNT bundle is wrapped many times around the fiber, which mitigates against delamination. By increasing the bias angle used during sheet wrapping (defined as the angle between the sheet ribbon direction and the nylon fiber direction) and increasing the ribbon width, the weight percent of CNT in the CNT/nylon composite fiber can be increased at will (and reached a maximum 30 wt% in the present investigation). The presently used bias angle for sheet wrapping was ~45°.

**Figure 1:**
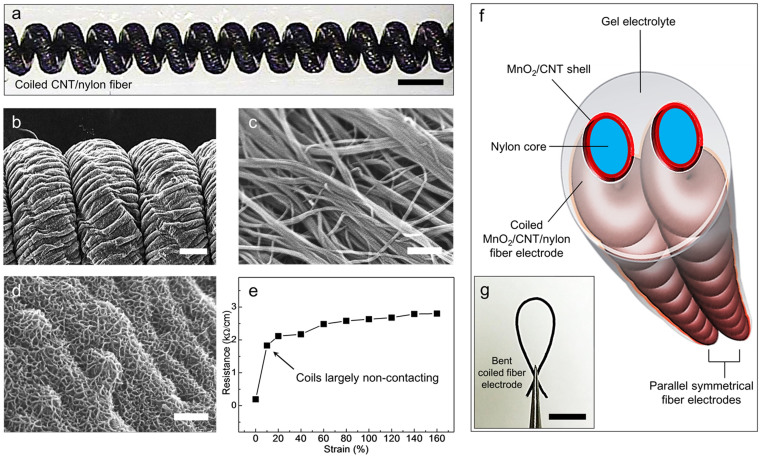
Coiled CNT/nylon electrode and electrode assembly into a supercapacitor. (a), Optical microscope image of a stretched, coiled CNT/nylon electrode fiber. This electrode was produced by helically wrapping a 102-μm-diameter nylon 6,6 monofilament fiber with a forest-drawn CNT sheet, and then highly twisting this composite fiber to produce complete fiber coiling (scale bar = 300 μm). (b), SEM image of a coiled CNT/nylon fiber electrode showing the wrinkles in the CNT layer that form during pre-coiling twist insertion, which increases the external surface layer that is available for electrolyte infiltration (scale bar = 50 μm). SEM images of the surfaces of CNT/nylon electrodes (c), before and (d), after mesoporous MnO_2_ deposition (scale bar = 150 nm for both c and d). (e), Resistance per length of the coiled CNT/nylon fiber electrode as a function of increasing applied strain. The separation of coils provides a major increase in resistance between 0 and ~10%, and there is a surprisingly small resistance change for further strain increase up to 150% elongation. (f), Schematic illustration for the complete all-solid-state supercapacitor, which comprises two symmetric coiled MnO_2_/CNT/nylon fiber electrodes and a gel electrolyte. (g), Optical image showing a bent MnO_2_/CNT/nylon fiber electrode, which is held by tweezers (scale bar = 5 mm).

While these mechanically robust CNT/nylon fibers can be used as non-elastic supercapacitor electrodes without further processing, we made these electrodes highly elastically deformable by inserting sufficiently high twist into them that they fully coil. Coiling started for a 102 μm diameter nylon fiber (under 8.73 MPa load relative to the nylon fiber cross-section) at ~2600 turns/m inserted twist (relative to initial fiber length), and an additional 1200 turns/m twist completed fiber coiling (Fig. 1a and Supplementary Fig. S2a). The initial fiber length decreased by 84% during twist insertion to obtain full coiling (and 15% prior to the start of coiling), which means that the completely coiled fiber can store more than 600% strain. The weight applied during coiling is the main parameter for controlling the coil index^8^, which is the ratio of coil diameter to fiber diameter (which was 2.3 for the present study).

This above contraction of the nylon fiber during pre-coiling twist insertion (by 15%) has two useful effects. First, the nylon diameter increases during pre-coiling twist insertion (so that the nylon fiber's density is approximately maintained during the length contraction), which tightens the bonding between the helically wrapped CNT sheet ribbon and the nylon fiber. Second, the 15% pre-coiling contraction in the length of the nylon fiber causes the wrapped sheet to form wrinkles that are approximately orthogonal to the nylon fiber direction in the coil, as shown in a scanning electron microscope (SEM) image of the coiled CNT/nylon fiber (Fig. 1b and Supplementary Fig. S2b, c). This wrinkling is expected to both increase electrolyte ion accessibility and provide reversible CNT array deformability as the substrate coils undergo giant elastomeric deformations.

Manganese dioxide (MnO_2_), which is promising pseudo-capacitive material because of its abundance in nature and environmental friendliness^14^, was electrochemically deposited onto the aligned CNT surface of coiled electrode (Fig. 1c) in order to enhance the electrochemical energy storage capacity of the electrode. As a consequence of the pseudo-capacitive contribution of the MnO_2_, the cyclic voltametry (CV) curve area for the electrode increased ~220% after MnO_2_ deposition (Supplementary Fig. S3a). X-ray photoelectron spectroscopy (XPS) analysis shows that the binding energy differences between Mn 2p_3/2_ and Mn 2p_3/2_ peaks of the deposit is about 11.8 eV (Supplementary Fig. S3b), which corresponds to that expected for MnO_2_^15^. As shown in the SEM micrograph of Fig. 1d, this electrochemical deposition of MnO_2_ results in MnO_2_ nanofiber chains which are separated by ~50 nm size pores. The weight of MnO_2_ introduced during electrochemical deposition was measured by using electrochemical quartz-crystal microbalance (EQCM)^16^. The thereby determined weight percent of MnO_2_ in the coiled, electrolyte-free electrode was about 1.45%, and the MnO_2_/CNT weight ratio was 5%.

Fig. 1e shows that the coiled CNT/nylon fiber electrode (before MnO_2_ coating) undergoes little change in electrical resistance over the strain region from ~10% to 150%. However, at low strains the electrode resistance abruptly increases with increasing strain, from 0.19 kΩ/cm at 0% strain to 1.83 kΩ/cm at below 10% strain. The origin of this conductivity decrease is the progressive elimination of inter-coil contact, which forces electronic carriers to loop along the coils, rather than additionally directly transporting between adjacent contacting coils. This conductivity decrease could be minimized by using an optimized small bias angle for sheet wrapping, selected to maximize nanotube orientation in the local nylon fiber direction of the coiled fiber. Nevertheless, the small change in resistance per fiber length on going from ~10% strain (1.8 kΩ/cm) to 150% strain (2.8 kΩ/cm) is remarkable, and probably results from progressive nanotube orientation towards the local electronic transport direction with increasing large strain.

Fig. 1f schematically illustrates the configuration of a complete all-solid-state, stretchable, coiled-fiber-electrode supercapacitor. Two identical coiled MnO_2_/CNT/nylon electrodes were placed parallel and coated with an aqueous PVA gel containing LiCl to complete the assembly of the highly stretchable supercapacitor. After this coating with electrolyte, the complete free-standing coiled fiber electrode was mechanically stable with respect to untwist (Fig. 1g).

### Electrochemical characterization of MnO_2_/CNT/nylon fiber supercapacitor

**Figure 2:**
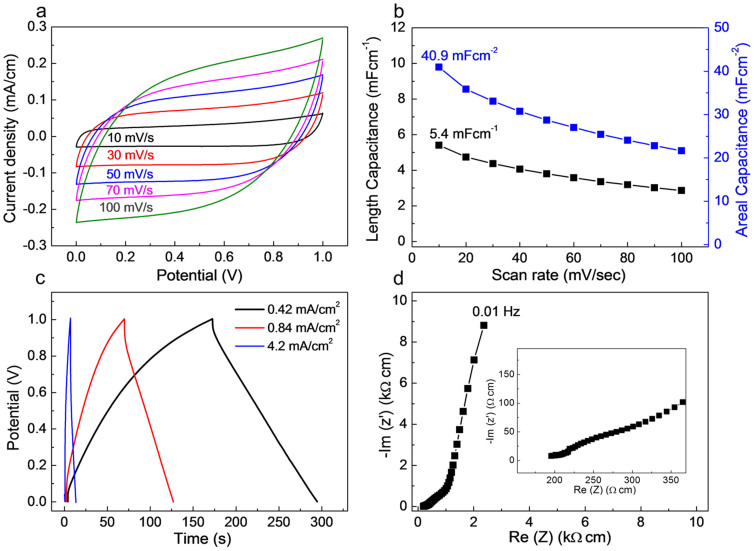
Electrochemical performance of the solid-state MnO_2_/CNT/nylon fiber supercapacitor. (a), CV curves measured from 10 to 100 mV/s for a solid-state MnO_2_/CNT/nylon fiber supercapacitor. The supercapacitor consists of two symmetrical coiled electrodes coated by PVA-LiCl gel electrolyte. (b), Calculated linear capacitance (normalized by supercapacitor length) and areal specific capacitance (normalized by the surface area of the MnO_2_/CNT/nylon electrode) as a function of voltage scan rate. (c), Galvano-static charge/discharge curves measured for from 0.42 mA/cm^2^ to 4.2 mA/cm^2^ current densities. (d), Nyquist curve for the frequency range from 0.01 to 1 kHz (the inset shows the high frequency region). The length of the supercapacitor for Fig. 2 measurements was 1 cm.

The electrochemical energy storage performance of the solid-state MnO_2_/CNT/nylon fiber supercapacitor was next evaluated. Fig. 2a shows CV curves measured for various scan rates from 10 to 100 mV/s. The pictured box-like rectangular CV curves, without any Faradic redox peaks, are consistent with an energy storage process that uses a combination of the electrochemical double-layer capacitance (EDLC) of the CNTs and the pseudo-capacitance of MnO_2_. Length-normalized and areal specific capacitances of single electrode are plotted in Fig. 2b. The highest values of the length- and area-normalized specific capacitances are 5.4 mF/cm and 40.9 mF/cm^2^, respectively, at a voltage scan rate of 10 mV/s, where the length and surface area of the electrochemically active materials (presently the MnO_2_/CNT/nylon electrodes) were used for normalization. This length-normalized capacitance of the coil supercapacitor is significantly higher than for previously reported for MnO_2_/CNT fiber supercapacitors (0.015 mF/cm)^4^ and ZnO nanowire/MnO_2_ based fiber supercapacitor (0.04 mF/cm)^17^, CNT spring fiber supercapacitor (0.51 mF/cm)^13^ and about the same as for a wet-spun CNT/graphene composite yarn supercapacitor (5.3 mF/cm)^6^. In addition, volume-normalized specific capacitance of the coil supercapacitor is 3.8 F/cm^3^ at 10 mV/s, which is smaller than CNT spring fiber supercapacitor (18.12 F/cm^3^)^13^, but still higher than MnO_2_/carbon fiber supercapacitor (2.5 F/cm^3^)^18^.

Galvanostatic charge-discharge curves for various current densities are presented in Fig. 2c, which are consistent with quasi-capacitive performance^19^ for the MnO_2_/CNT/nylon fiber supercapacitors. From electrochemical impedance spectroscopy (EIS) measurements (Fig. 2d), the normalized equilibrium series resistance (ESR) at 1 kHz for the solid-state supercapacitor is 217 Ω cm. CV scans for a solid-state supercapacitor comprising two symmetrical MnO_2_/CNT/nylon fiber electrodes are provided for different voltage ranges (Supplementary Fig. S4a) and different voltage scan rates (Supplementary Fig. S4b). The box-like stable CV curves with large potential window of 1.4 V could be obtained from two-symmetric electrode system.

### Static and dynamic capacitances of the fiber supercapacitor during large-stroke deformations

**Figure 3:**
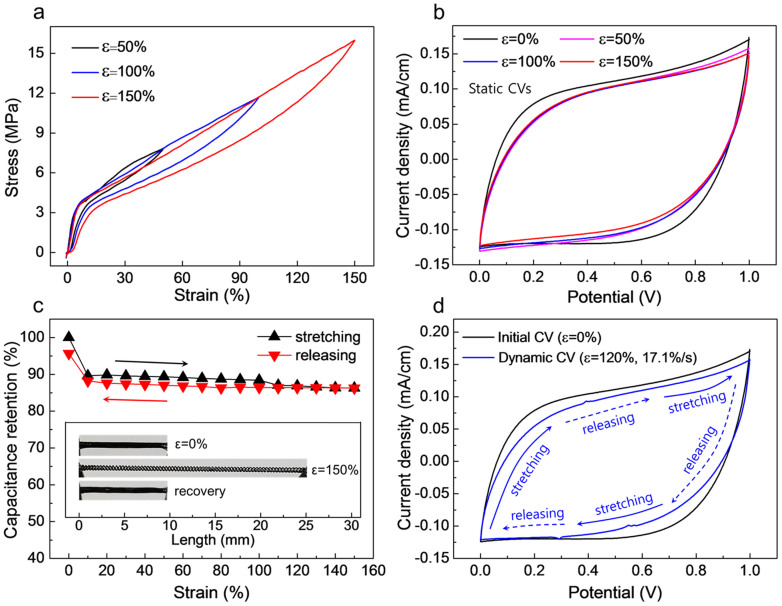
Static and dynamic capacitances of the solid-state fiber supercapacitor during large-stroke tensile deformations. (a), Stress loading-unloading curves for a MnO_2_/CNT/nylon gel-coated single fiber electrode for 50 to 150% maximum applied tensile strain. (b), CV curves measured for the initial state (ε = 0%) and statically stretched states (ε = 50, 100, 150%) are compared, where the potential scan rate is 50 mV/s. (c), Capacitance retention is plotted versus applied strain during a stretch-release cycle up to 150% strain (the inset shows optical images of complete coil supercapacitor before, at, and after 150% strain for the capacitance retention test). Capacitances were derived from the CV curves for the potential range between 0 and 1 V, using a voltage scan rate of 50 mV/s. (d), Dynamic CV curve (scan rate of 50 mV/s) measured during stretch/release cycles to 120% strain at a strain rate of 17.1%/s (blue line). The initial CV curve (black line) for the unstrained supercapacitor is presented for comparison.

The elasticity of the coiled electrode MnO_2_/CNT/nylon solid-state supercapacitor is evaluated in Fig. 3a, which shows that at stretch/release cycle to 150% tensile strain results in less than a 5% residual deformation. At low strains (below 6% strain), where the coils are contacting, the Young's modulus for stretch of the solid-state supercapacitor in the fiber electrode direction is high (~68 MPa). At higher strains, where the coils are no longer contacting, the Young's modulus of the supercapacitor abruptly decreases to ~8.7 MPa, and stays at this value up to at least 150% strain. After this ~150% reversible region, where coil separation is the dominant mechanism enabling stretchability, the supercapacitor could be partially irreversibly elongated to 500% strain (which causes coil straightening). Hysteresis during a stretch/stretch release cycle to 150% strain shows 18.4% energy loss. Similar hysteretic stress/strain curves have been observed for coiled carbon nanotube yarns^20^.

The application of static tensile strains (Fig. 3b) results in only a minor change in CV curves for up to 150% applied tensile strain. As shown here, while CV curves for 50%, 100% and 150% strain nearly superimpose, the CV curve for 0% strain corresponds to a slightly higher current density for a given potential (i.e., a slightly higher effective capacitance) than for the CV curves for higher applied tensile strains of 50 to 150% strain, which is likely due to the transition from contacting to non-contacting coils that occurs at below 10% strain (and is seen in the stress-strain curves of Fig. 3a for the solid-state supercapacitor, as well as in the linear resistance versus strain curve of Fig. 1e for a single coiled CNT/nylon electrode fiber). Fig. 3c shows that the dependence of capacitance on strain is largely independent of strain for above 10% applied strain, and that the capacitance measured for increasing strain nearly coincides with that obtained for decreasing strain (except for a 5% deviation at below 10% strain). Also, the Fig. 3c inset shows that the solid-state supercapacitor returns to its original length after stress is released from 150% strain.

While the above results show that static stretch to 150% strain little effects the capacitance of the solid-state supercapacitor, it is also important to demonstrate that dynamically applied cyclic strains during capacitor charge and discharge do not degrade supercapacitor performance^21^. The combined data of Fig. 3d and Supplementary Fig. S5 shows that the CV curve of a MnO_2_/CNT/nylon fiber supercapacitor (and therefore the capacitance of the supercapacitor) is little affected by repeated cycling from 0% to 120% strain during the CV cycle. The capacitance retention, relative to the capacitance for 0% strain, was 86.5, 90.8, and 87.8% for engineering strains of 6, 12, and 17.1% strain/s, respectively. Although the separation between parallel coiled MnO_2_/CNT/nylon fiber electrodes in the solid-state supercapacitor was only ~100 μm, there was no tendency for short-circuiting of the 1 cm- long capacitor electrodes during stretch.

### Energy and average power densities for the complete stretchable fiber supercapacitor

**Figure 4:**
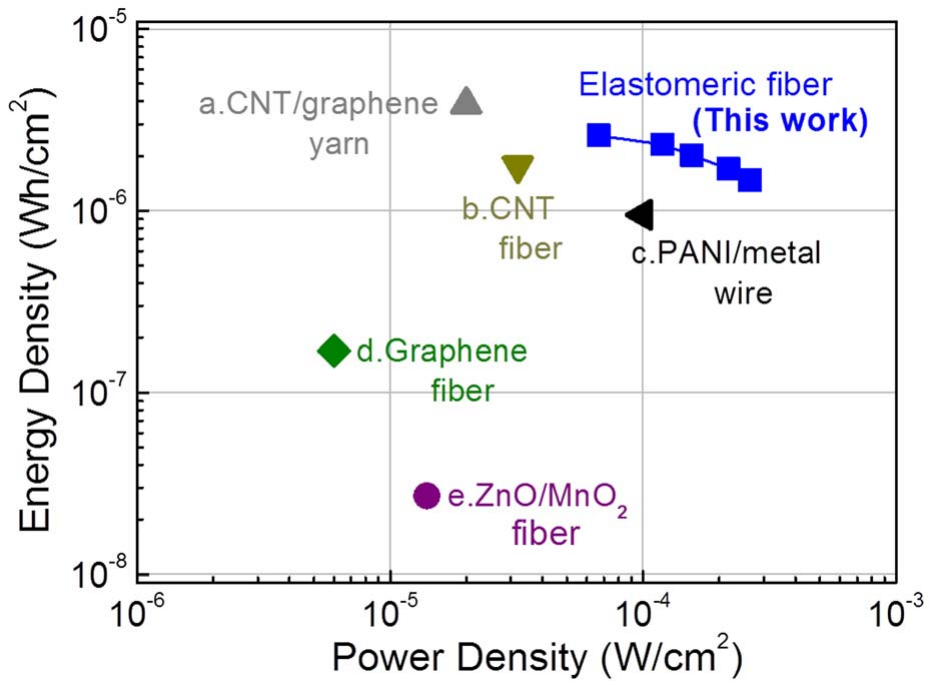
Areal energy and average power densities for complete fiber supercapacitors. A Ragone plot of areal energy density versus areal power density for a highly elastomeric coiled MnO_2_/CNT/nylon fiber supercapacitor (based on the total surface area of the complete supercapacitor) is compared with the published performance of flexible yarn and fiber supercapacitors (none of which offer the benefits of reversible elastomeric deformability). The maximum measured energy density of the presently investigated supercapacitor is 2.6 μWh/cm^2^, which is comparable to that for (a), the flexible wet-spun CNT/graphene yarn supercapacitor (3.84 μWh/cm^2^)^6^. This areal capacitance of the present solid-state supercapacitor is higher than for other flexible fiber supercapacitors: (b), a mesoporous carbon/CNT fiber supercapacitor (1.77 μWh/cm^2^)^22^, (c), a PANI/stainless steel wire supercapacitor (0.95 μWh/cm^2^)^23^, (d), a graphene fiber supercapacitor (0.17 μWh/cm^2^)^24^, and (e), a ZnO nanowire/MnO_2_ fiber supercapacitor (0.027 μWh/cm^2^)^17^.

Using the Supplementary Fig. S4b results for 1.4 V charge/discharge, energy and power densities were obtained, as well as the dependence of areal energy density on areal power density, which is shown in the Ragone plot (Fig. 4). The maximum measured energy density of the presently investigated stretchable supercapacitor is 2.6 μWh/cm^2^, which is comparable to that for the flexible wet-spun CNT/graphene yarn supercapacitor (3.84 μWh/cm^2^)^6^. This areal density of the present solid-state stretchable supercapacitor is higher than for other flexible fiber supercapacitors: a mesoporous carbon/CNT fiber supercapacitor (1.77 μWh/cm^2^)^22^, a PANI/stainless steel wire supercapacitor (0.95 μWh/cm^2^)^23^, a graphene fiber supercapacitor (0.17 μWh/cm^2^)^24^, and a ZnO nanowire/MnO_2_ fiber supercapacitor (0.027 μWh/cm^2^)^17^. Such areal figures of merit (based on surface area of the complete solid-state fiber supercapacitor, including the gel electrolyte coating) are here emphasized since they determine the maximum energy density and energy storage capacity that can be obtained from a given area of woven textile.

### Demonstration of woven fiber supercapacitors

**Figure 5:**
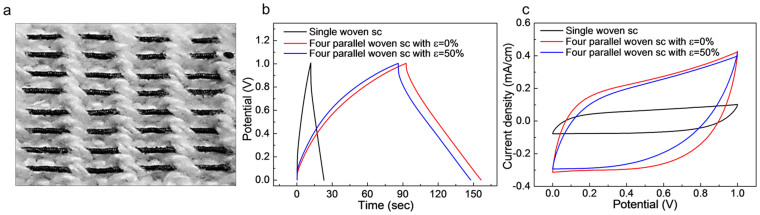
Woven fiber supercapacitor and its electrochemical performances. (a), Optical image showing 1.5 cm long eight MnO_2_/CNT/nylon fiber electrodes woven into the wristband of the glove. (b), Galvanostatic-charge/discharge curves and (c), CV curves (50 mV/s scan rate) of the woven supercapacitor (sc) are shown. The performances of single supercapacitor consisting of two woven fiber electrodes (black line), four parallel connected fiber supercapacitors before (red line) and after 50% strain stretching (blue line) are compared in (b and c), respectively.

To demonstrate the weavability, eight elastomeric MnO_2_/CNT/nylon electrodes were sewn into the wristband of the glove (Fig. 5a and Supplementary Fig. S6a) and connected by Cu wires for electrical connection (Supplementary Fig. S6b). Four supercapacitors, each comprising two fiber electrodes, were connected in parallel direction and over coated by solid-state electrolyte for complete woven supercapacitor. The charge/discharge and CV curves of single and four parallel connected fiber supercapacitors were measured as shown in Fig. 5b and c. After 50% strain application in coil length direction, minor changes in charge/discharge time and CV area were obtained, compared with not deformed woven supercapacitors.

## Discussion

Since we have deployed the utilized the twist-insertion-based coiling method to obtain highly elastomeric behaviors for other diverse high strength monofilament and multifilament polymer fibers (like polyethylene, Kevlar, silver-plated nylon 6, polyester, polypropylene, and polyvinylidene difluoride)^8^, the presently described supercapacitor fabrication method can be extended to supercapacitors that operate at high temperatures and use either hydrophilic or hydrophobic electrolytes.

## Methods

### Preparation of coiled electrodes

MWNT sheets were drawn from a CNT forest (~400 μm high and consisting ~12 nm diameter nanotubes containing ~9 walls), which was fabricated by chemical vapor deposition using the previously reported method^9^. A 102 μm diameter commercially available nylon 6,6 fiber (Coats and Clark D67 4 mil) was used for electrode fabrication. After helically wrapping the CNT sheet ribbons on the nylon fibers, the CNT/nylon fiber were twisted ~3,800 turns per meter to make the coil fiber electrode. One end of each electrode was connected to 180 μm diameter Cu wire by using silver paste. The electrochemical deposition of MnO_2_ was performed on the coiled CNT/nylon fiber electrode using the potentiostatic method by applying 1.3 V (vs. Ag/AgCl reference electrode) while Pt mesh was used as the counter electrode. The electrolyte used for electrodeposition contained 0.02 M MnSO_4_·5H_2_O and 0.2 M Na_2_SO_4_.

### Supercapacitor assembly

The PVA-LiCl gel electrolyte was prepared by mixing 3 g PVA (*M_w_* 146,000 ~ 186,000) and 6 g LiCl in 30 ml deionized water and heating this mixture at 90°C for several hours until it become transparent. Two symmetrical coiled MnO_2_/CNT/nylon fiber electrodes were placed parallel (with a small gap of ~100 μm) and coated by the PVA-LiCl gel electrolyte to complete fabrication of the fiber supercapacitor. All chemicals for electrolyte synthesis were purchased from Sigma-Aldrich.

### Characterization

All static electrochemical measurements on the fiber supercapacitor utilized a two-electrode configuration and a electrochemical analyzer (CHI 627b, CH Instrument). Dynamic measurements of CV curves were made on a fiber supercapacitor by cycling at 50 mV/s, while the supercapacitor was stretched and released at set strain rates of 6, 12, and 17.1%/s (by using a specially constructed machine for applying tensile deformations). The length of the supercapacitor was measured using a digital vernier-caliper (500 series, Mitutoyo) which was incorporated into the stretching machine. Stress loading-unloading curves were obtained by using mechanical analyzer (Instron 5966). SEM images were obtained by using a Hitachi SEM-S4700 microscope.

### Calculation of the electrochemical performances

The capacitance for two-electrode system was calculated from CV curves. From C = I/(dV/dt), where I is discharge current, the single-electrode specific capacitance (C_sp_) was calculated from the following equation.
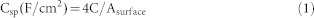
where A_surface_ is the total surface area of the electrochemically active materials of two electrodes (MnO_2_/CNT/nylon coil electrodes for present work). Total length and volume of active materials were used for linear and volumetric capacitance calculation, respectively. The factor of 4 comes from the capacitance of the two-electrode system and the combined volume of two electrodes^7^.

For a given constant scan rate ν and initial discharge voltage (V_i_), the average power was calculated by integrating the current (I) versus voltage (V) curves^1^;
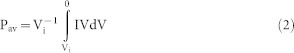
The discharged energy was calculated by using equation (3);
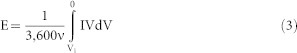


Resulting areal energy and average power densities for the complete supercapacitor (using total surface area of the supercapacitor including both MnO_2_/CNT/nylon coil electrodes and solid electrolyte) are indicated in Fig. 4.

## Author Contributions

C.S.C. and S.J.K. conceived the idea and designed the experiments; S.H.K., H.J.S. and J.A.L. contributed mechanical/electrochemical characterization; A.Y.C. and Y.T.K. analyzed data; X.L. fabricated material for experiments; C.S.C., S.J.K., G.M.S. and R.H.B. wrote the paper. All authors discussed the results and commented on the manuscript.

## Supplementary Material

Supplementary Information SUPPLEMENTARY INFO
