# Supplementary material for: Stretchable, Weavable Coiled Carbon Nanotube/MnO2/Polymer Fiber Solid-State Supercapacitors
Source: Sci Rep. 2015 Mar 23;5:9387. doi: 10.1038/srep09387 (PMC4369743; doi:10.1038/srep09387)
Supplement: Supplementary Information — SUPPLEMENTARY INFO [file srep09387-s1.pdf]

SUPPLEMENTARY INFORMATION

Stretchable, Weavable Coiled Carbon Nanotube/MnO_2/Polymer Fiber Solid-State Supercapacitors

Changsoon Choi[1], Shi Hyeong Kim[1], Hyeon Jun Sim[1], Jae Ah Lee[1], A Young Choi[2], Youn Tae Kim[2], Xavier Lepró[3], Geoffrey M. Spinks[4], Ray H. Baughman[3] and Seon Jeong Kim[1]*

[1] C. Choi, S.H. Kim, H.J. Sim, J.A. Lee, Prof. S. J. Kim
Center for Bio-Artificial Muscle and Department of Biomedical Engineering,
Hanyang University, Seoul 133-791, Korea
[*] E-mail: sjk@hanyang.ac.kr

[2] A. Y. Choi, Prof. Y. T. Kim
IT Fusion Technology Research Center and Department of IT Fusion Technology,
Chosun University, Gwangju 501-759, Korea

[3] Dr. X. Lepró, Prof. R. H. Baughman
The Alan G. MacDiarmid NanoTech Institute, University of Texas at Dallas, Richardson, TX 75083, USA

[4] Prof. G. M. Spinks
Intelligent Polymer Research Institute, ARC Centre of Excellence for Electromaterials Science, University of Wollongong, Wollongong, NSW 2522, Australia

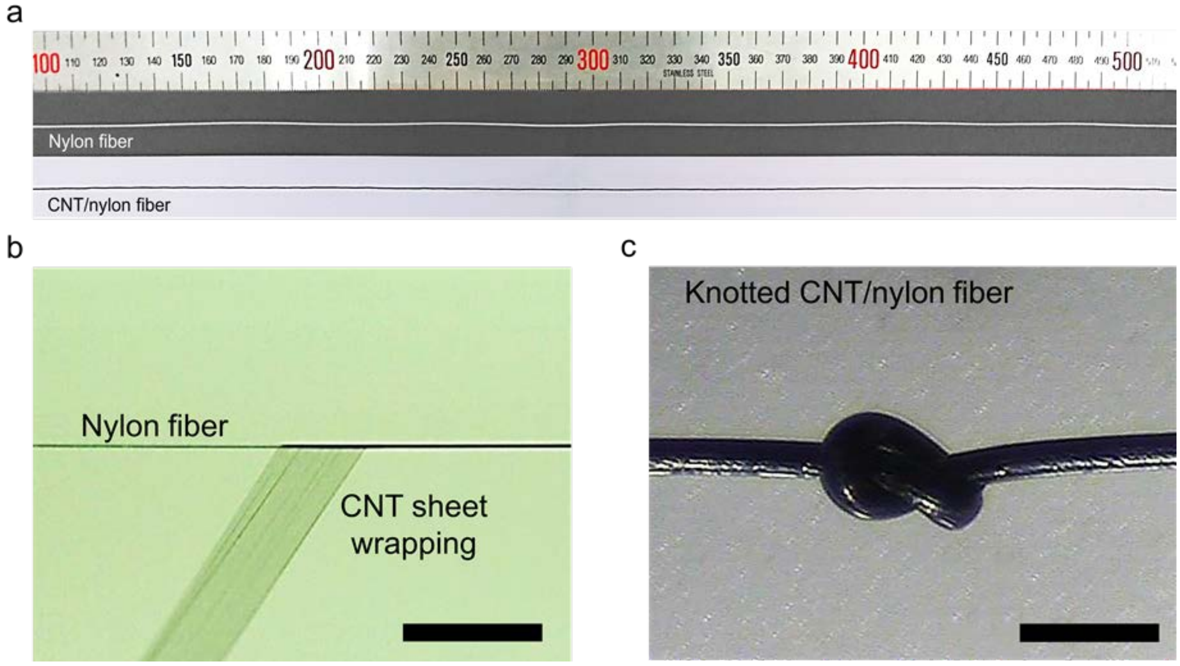

Fig. S1. Photographs of **(a)** the nylon 6,6 fiber before (top) and after (bottom) CNT sheet wrapping and **(b)** a nylon fiber during helical wrapping with a CNT sheet. The nylon fiber was rotated as the CNT forest (from which the CNT sheet was automatically drawn) was translated parallel to rotation axis of the fiber (so as to maintain a constant angle between the nylon fiber and CNT sheet and provide uniform sheet wrapping). **(c)** Photograph of a knotted, CNT-wrapped nylon fiber. Before knotting, the wrapped CNT sheet on the nylon fiber was densification by dropping ethanol onto the fiber surface and allowing ethanol evaporation. After solvent densification, the CNT coating was strongly attached to the nylon fiber, which enabled fiber knotting without delamination of the helically wrapped CNT sheet. The scale bars of (b) and (c) are 1 cm and 500 μm, respectively.

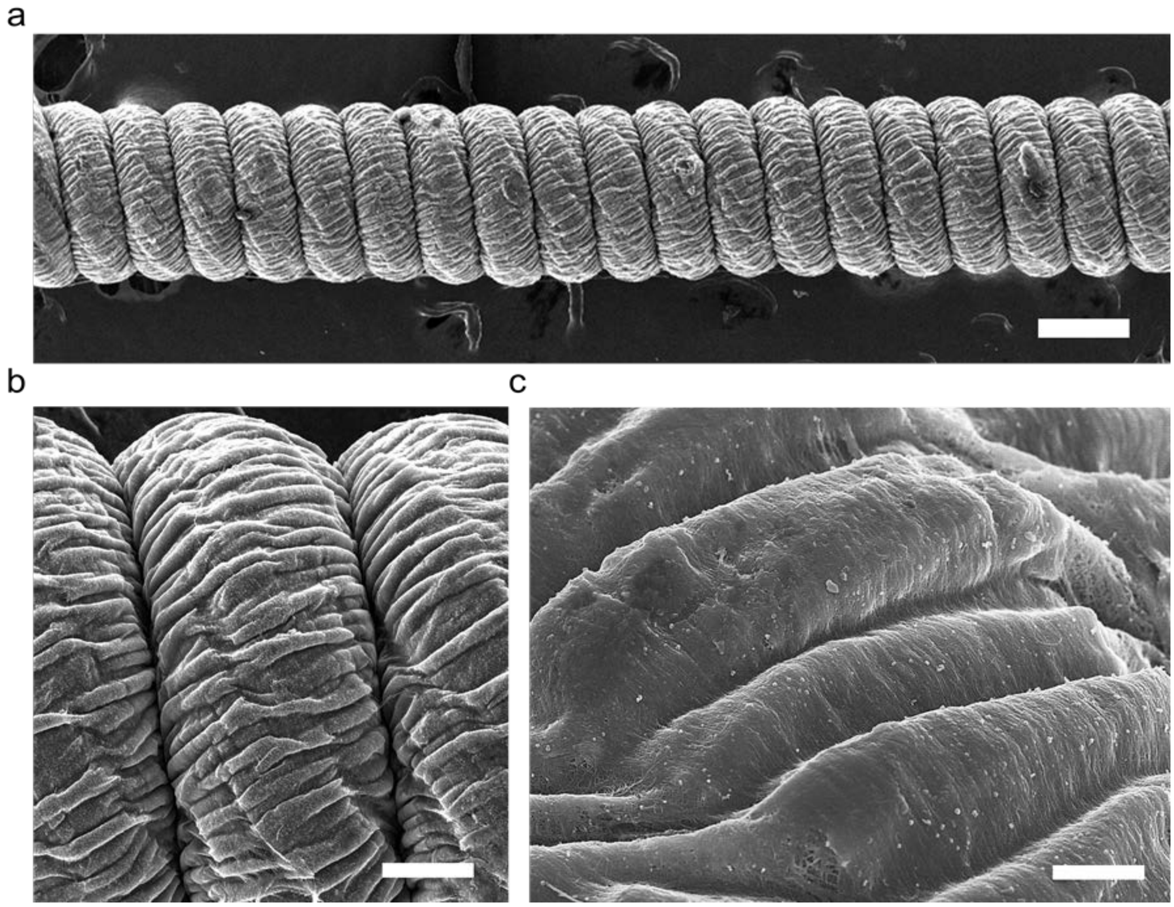

Fig. S2. SEM images of a coiled CNT-wrapped nylon fiber at low **(a)**, middle **(b)**, and high **(c)** magnifications. The scale bars are 200 μm, 50 μm, and 10 μm, respectively. The pictured wrinkling of the CNT layer results from contraction of the fiber length during twist insertion, which is accompanied by ~18% increase in fiber diameter.

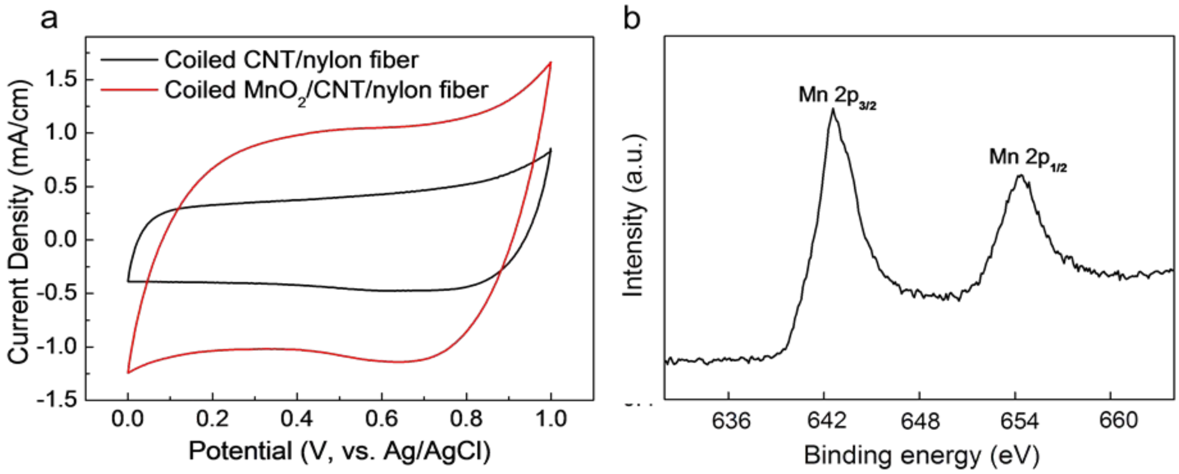

Fig. S3. (a) Comparison of CV curves before and after MnO_2 deposition on a coiled CNT/nylon fiber. The curves were measured in three-electrode system, using Pt mesh as counter electrode and a Ag/AgCl reference electrode. **(b)** XPS analysis of Mn peaks. The binding energy difference between $Mn_{2p3/2}$ and $Mn_{2p3/2}$ is 11.8 eV, which corresponds to expectations for MnO_2 [15]

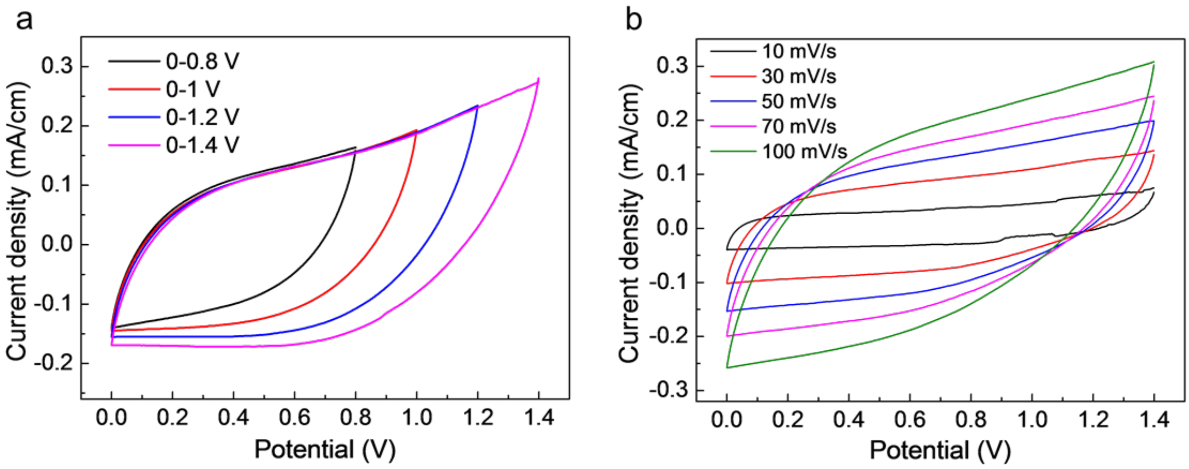

Fig. S4. CV curves for a solid-state supercapacitor comprising two parallel symmetric coiled MnO_2/CNT/nylon fibers that are jointly coated with a PVA/LiCl gel electrolyte. **(a)** CV curves measured using different voltage scan ranges and a voltage scan rate of 50 mV/sec. **(b)** CV curves from 0 to 1.4V for various scan rates from 10 to 100 mV/sec. All measurements are performed in two-electrode system.

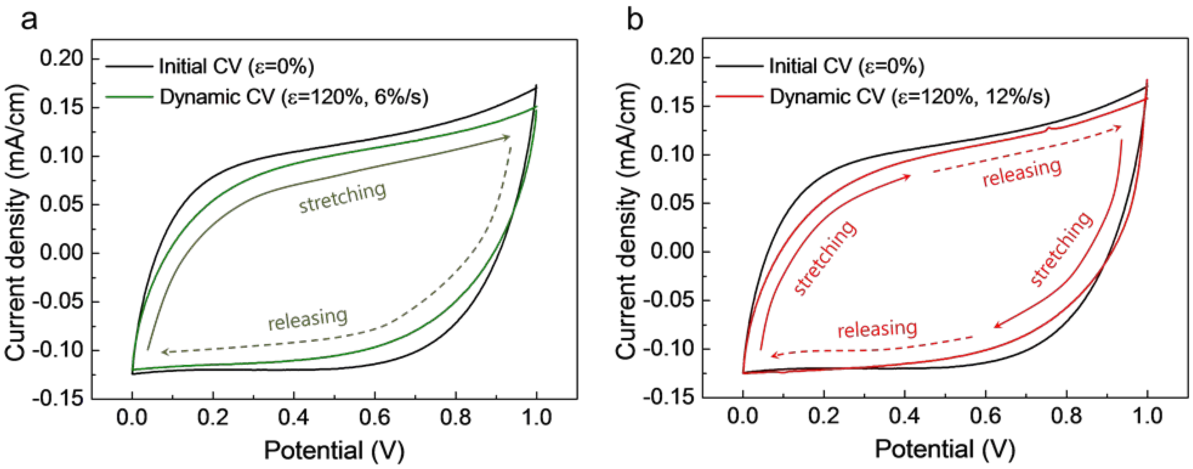

Fig. S5. Dynamic CV curve (scan rate of 50 mV/s) measured during real-time stretch/release cycles to 120% strain at a scan rate of **(a)** 6%/s (green line) and **(b)** 12%/s (red line). The CV curve (black line) for the initial unstrained supercapacitor is presented for comparison in both (a) and (b). The percentage retention of the CV area for the unstrained supercapacitor was ~86.5% for a mechanical strain rate of 6%/s and 90.8% for a strain rate of 12%/s.

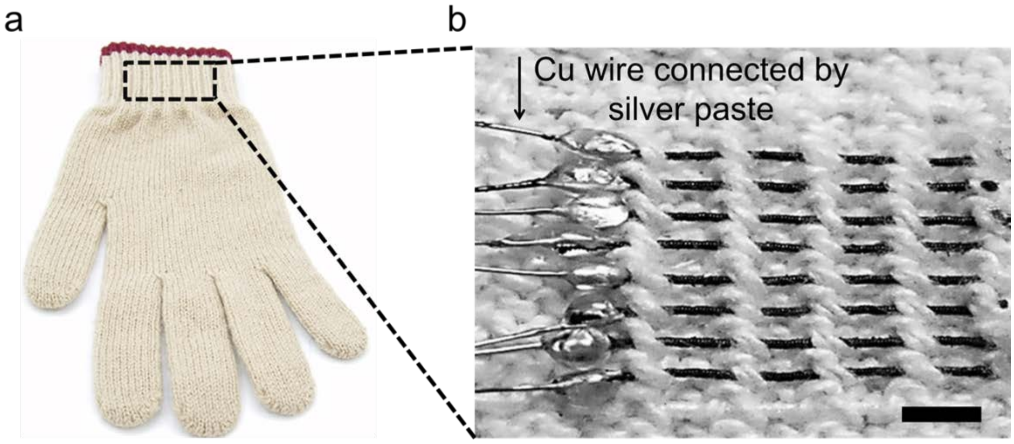

Fig. S6. Optical images of **(a)** the glove used for demonstration of sewn supercapacitors and **(b)** the inner side of the glove wristband, showing eight sewn supercapacitor electrodes and the electrical connections used to deploy these electrodes in four supercapacitors. After sewing the electrodes into the glove wristband, the electrode array was over-coated with the gel electrolyte. The scale bars for image (b) is 3 mm.
